# Detection and characterization of spike architecture based on deep learning and X-ray computed tomography in barley

**DOI:** 10.1186/s13007-023-01096-w

**Published:** 2023-10-27

**Authors:** Yimin Ling, Qinlong Zhao, Wenxin Liu, Kexu Wei, Runfei Bao, Weining Song, Xiaojun Nie

**Affiliations:** 1 State Key Laboratory of Crop Stress Biology in Arid Areas and College of Agronomy, Northwest A&F University, Yangling, 712100 Shaanxi China; 2 ICARDA-NWSUAF Joint Research Centre, Northwest A&F University, Yangling, 712100 Shaanxi China

**Keywords:** Barley, 3D morphological processing, Computational modeling, Spike architecture

## Abstract

**Background:**

Spike is the grain-bearing organ in cereal crops, which is a key proxy indicator determining the grain yield and quality. Machine learning methods for image analysis of spike-related phenotypic traits not only hold the promise for high-throughput estimating grain production and quality, but also lay the foundation for better dissection of the genetic basis for spike development. Barley (*Hordeum vulgare* L.) is one of the most important crops globally, ranking as the fourth largest cereal crop in terms of cultivated area and total yield. However, image analysis of spike-related traits in barley, especially based on CT-scanning, remains elusive at present.

**Results:**

In this study, we developed a non-invasive, high-throughput approach to quantitatively measuring the multitude of spike architectural traits in barley through combining X-ray computed tomography (CT) and a deep learning model (UNet). Firstly, the spikes of 11 barley accessions, including 2 wild barley, 3 landraces and 6 cultivars were used for X-ray CT scanning to obtain the tomographic images. And then, an optimized 3D image processing method was used to point cloud data to generate the 3D point cloud images of spike, namely ‘virtual’ spike, which is then used to investigate internal structures and morphological traits of barley spikes. Furthermore, the virtual spike-related traits, such as spike length, grain number per spike, grain volume, grain surface area, grain length and grain width as well as grain thickness were efficiently and non-destructively quantified. The virtual values of these traits were highly consistent with the actual value using manual measurement, demonstrating the accuracy and reliability of the developed model. The reconstruction process took 15 min approximately, 10 min for CT scanning and 5 min for imaging and features extraction, respectively.

**Conclusions:**

This study provides an efficient, non-invasive and useful tool for dissecting barley spike architecture, which will contribute to high-throughput phenotyping and breeding for high yield in barley and other crops.

**Supplementary Information:**

The online version contains supplementary material available at 10.1186/s13007-023-01096-w.

## Background

The global food demand is expected to increase by 35 to 56% from 2010 to 2050, and how to meet this demand is uncertain [1]. Due to climate change, population booming as well as following the COVID-19 pandemic, it has great challenges in regard to total food production, food supply chains and food security [2]. It is reported that global food production must increase by 1.5% annually to fulfill those needs, especially grain-related cereal crop production [3]. Consequently, it is necessary to expedite the genetic improvement of crops for breeding of new cultivars with high yield, desired characteristics and strong resistance to diverse stresses [4].

Barley (*Hordeum vulgare* L.) is the world’s fourth-largest cereal crop in terms of cultivation area and total grain yield after maize, rice and wheat, which is also one of the earliest domesticated crops to contribute significantly to agriculture and human civilization [5]. Predominantly, barley has important edible, feeding, and industrial brewing value [6]. The inflorescence of barley and wheat displays a raceme-like branchless shape called spike, as the grain-bearing organ, which is the primary source of grains that are harvested. Thus, better dissection of the spike architecture and high throughput phenotyping of spike-related traits will contribute to better understanding the genetic basis for spike development and yield formation, and then accelerate the breeding for high-yield varieties.

Nowadays, gene chip and deep sequencing technologies represent the commonplace high-throughput genotyping approach to quickly and efficiently obtaining genotypic information in barley [7, 8]. While for phenotyping, visual observation is still the commonly used technique for assessing barley spike features, although optical sensing is increasingly adopted as a quicker and more precise method for collecting phenotypic data [9]. Photons can be scattered and absorbed by plant tissues, resulting in reflection, transmission, and absorption which can be measured via optical sensing [10]. Many sensing methods have been developed to monitor the physical and chemical characteristics of plants, such as X-ray computed tomography (CT), red–green–blue (RGB) imaging, and chlorophyll fluorescence (ChlF) [11]. Utilizing X-ray CT technology, the projection of morphological features can be visualized by recognizing discrepancies in energy absorption from a variety of perspectives before and after scanning [12]. Several studies have revealed that X-ray CT scanning provides a non-destructive and efficient approach to perceiving the 3D inner and outer structures of plant samples with improved accuracy [13]. Hughes et al. conducted the precise extraction and quantification of wheat spike characteristics and grain morphological features through X-ray microscopic CT, demonstrating that CT scanning was far more expeditious than manual appraisal, in addition to being non-invasive [14]. A similar method based on X-ray CT scans was also developed for characterizing rice panicle traits [12]. Additionally, X-ray CT was used to evaluate panicle morphology in sorghum, revealing continuous morphological variation across genetically diverse germplasm [15].

Although X-ray CT imaging is already employed for high-throughput phenotyping in some crop species, its efficiency could be improved by leveraging deep learning models [14, 15]. In the past, X-ray CT data was processed using conventional computer vision means, such as threshold segmentation by image binarization, which are not capable of handling a high amount of interference. Artificial intelligence technique, such as deep learning (DL), which is a subdivision of machine learning (ML), can be employed to cull features from massive amounts of data for a variety of intricate tasks [16]. DL technology is widely used in automating and addressing high-throughput phenotyping [17], crop yield prediction [18], optimizing irrigation [19], AI-based soil chemical analysis and fertilization [20], crop disease mapping and management [21], and crop optimization and modeling [22]. To address biomedical image segmentation quandaries, Ronneberger et al. proposed a UNet model that was developed based on a fully convolutional neural network (FCN) [23], which has been extensively applied to segmentation in CT images, especially in the medical field [24], including COVID-19 and other diseases [25] and segmentation of infected tissues [26].

However, X-ray CT segmentation is rarely used in plants due to many CT hardware and software have been designed for medical applications. Optimized X-ray CT workflow for botanical study purposes is crucial to addressing a range of phenotypic traits from plant organs. With emphasis on more accurate plant trait investigation, image analysis based on X-ray CT to automatically identify and segment regions of interest (ROI) in CT images has gradually increased [14]. CT analysis requires extensive processing whereby a stack of tomographic images must be segmented layer by layer [27]. Huges et al. rejoined the split spikes by Z axis after isolation of grain from slice images, which is based on 2D image processing and doesn’t fully make use of the 3D information [14]. Biao et al. proposed another effective method using 3D voxels and directly processing the 3D images in wheat [28]. Furthermore, Hu et al. used a similar 3D image method to isolate wheat spikelet from spike by finding the nearest grain [29]. Therefore, 3D image processing method provided a more effective and optimized method to measure plant phenotypic traits based on 3D morphology.

In this study, we integrated X-ray CT imaging technology and the UNet DL model to segment and quantify morphological characteristics of barley spikes and grains as well as their spatial distribution by point cloud 3D methods. The aim was to develop a novel, efficient and non-destructive way to phenotype barley spike-related traits, that could contribute to a better understanding of the genetic basis of spike development and eventually facilitate breeding of high yielding varieties in barley and other crops.

## Results

### Development of a pipeline of CT image processing with a robust DL-based segmentation for barley spikes

**Fig. 1:**
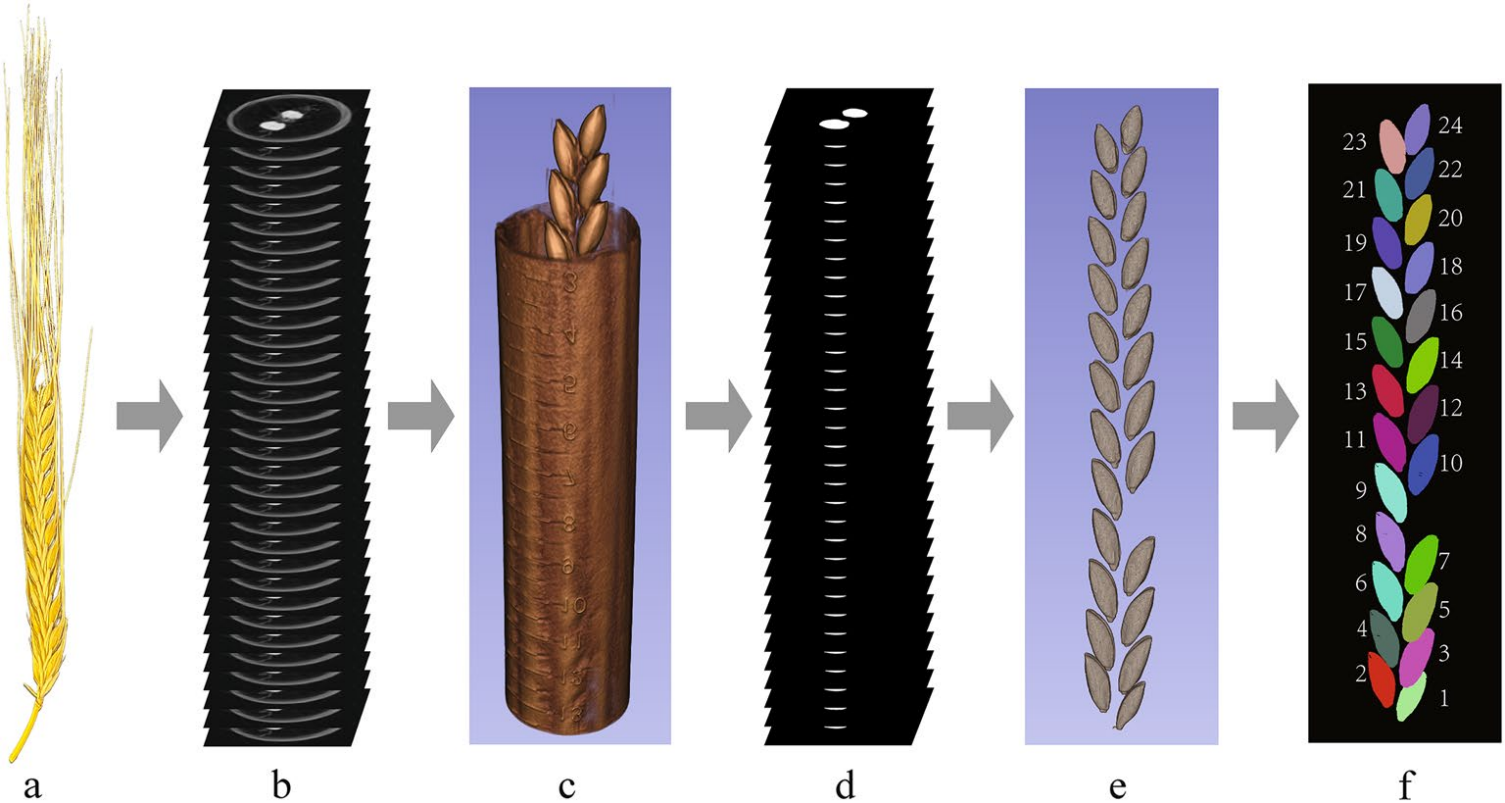
Pipeline of CT-based extraction of spike phenotypic traits of barley. **a** Optical image of a barley spike. **b** Slice by slice scanned CT images of a barley spike. **c** 3D reconstruction preview of CT images. **d** Slice by slice detection of region of interest (ROI) from CT images. **e** 3D reconstruction preview of ROI images. **f** Detection of barley grains by proposed 3D morphological analysis. The colors in the figures are randomly adopted

**Fig. 2:**
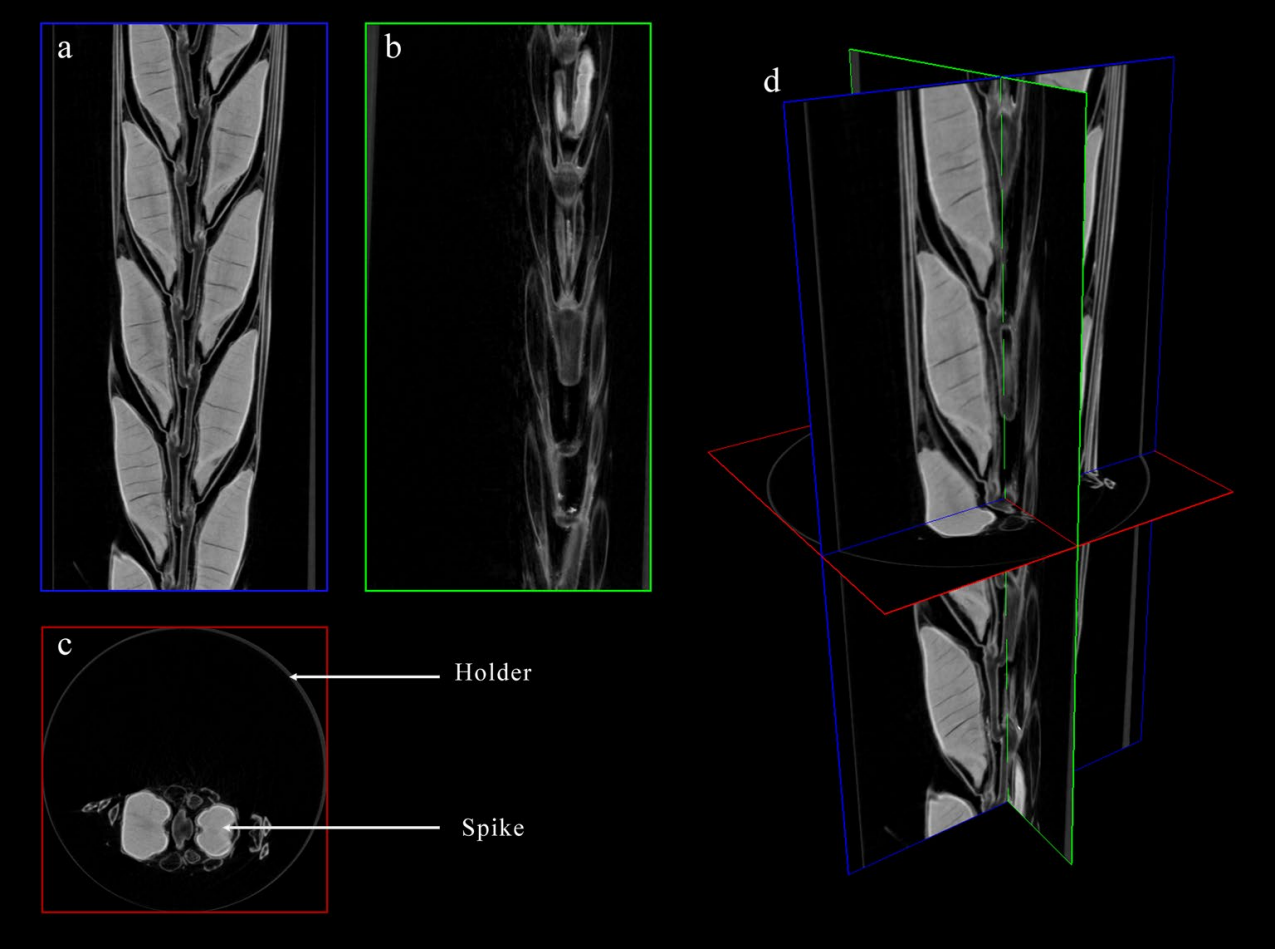
CT 3D reconstructed high resolution slice images of a barley spike (22 µm/pixel). **a** Coronal section. **b** Sagittal section. **c** Transverse section. **d** Three section stacked image

For high-throughput image analysis through deep machine learning, 3D reconstruction modeling is the initial and indispensable process, which necessitates the automatic batch processing of a considerable number of CT scans for the purpose of reconstructing the initial targeted item. To achieve high-throughput assessing the barley spike architecture and dissect spike-related traits based on CT tomography, a pipeline was developed to reconstruction the 3D modeling of the barley spike by integration of CT image processing and the robust DL-based segmentation (Fig. 1). First of all, the barley spikes were collected from the field and then naturally dehydrated. And then, they were fixed by a holder in the scanner for straightening and scanned at the maximum resolution (75–95 µm/pixel, Additional file 1: Table S1). The scanning could achieve covering all the grains in less than 10 min. The CT projection images were produced automatically by the scanner software and outputted as CT transaxial slice images (Fig. 2). A large dataset of 13,074 CT images was generated, and subsets were selected for phenotypic trait extraction and DL training (detailed images number in Additional file 2: Table S2). The slice images were processed into a stacked image format to conserve hardware space. The CT scanning produced a total of 7 gibibytes (GiB) of projection images, which were then compressed using processing scripts into 402 mebibytes (MiB) stacked images for extraction of phenotypic information.

**Fig. 3:**
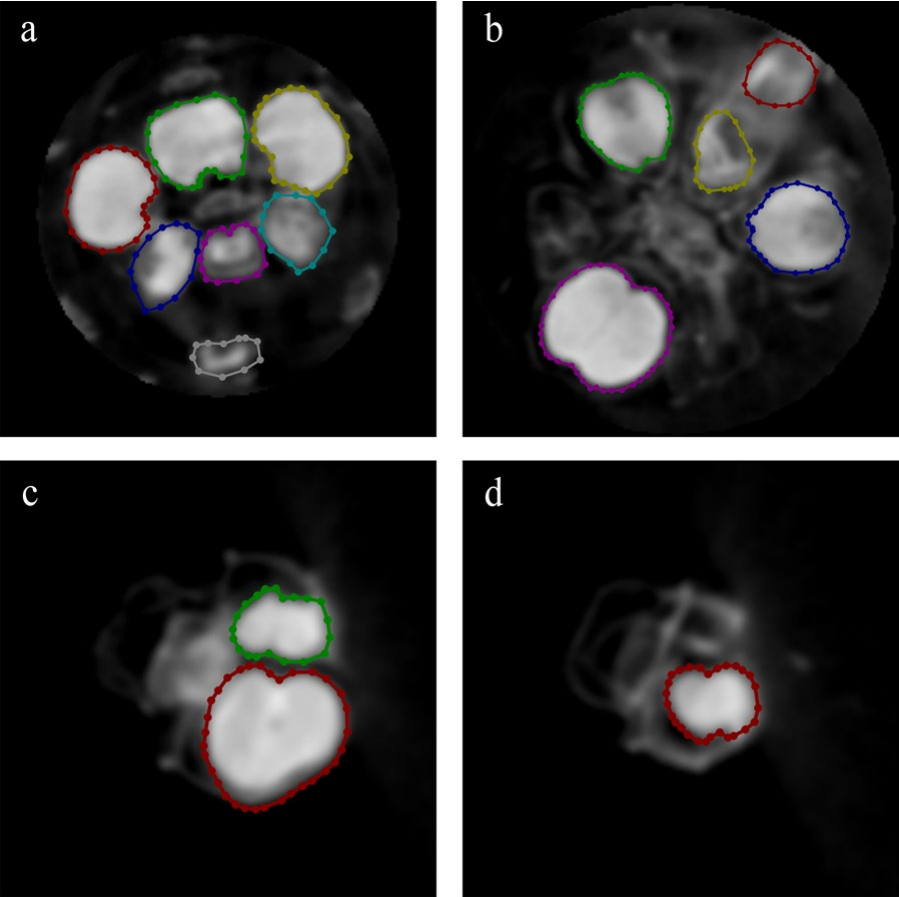
Samples of images of manually labeled regions of grains from a CT slice. **a**, **d** Labeled slice image of barley variety s17350. **b** Labeled slice image of wheat cultivar s113095. **c** Labeled slice image of wheat cultivar D3

The Manual delineation of ROI via visual assessment of CT images may be laborious and unreliable [30]. In order to tackle this issue of barley spike, we employed the classic UNet structure and developed a model to detect ROI in CT images. The model segmented the ROI based on DL training from raw slice images or pre-segmented slice images. We trained UNet models using 124 labeled training images, consisting of manually labeled CT images of barley variety s113095, s17350 and wheat cultivar D3 (Fig. 3). The other 30 labeled images were used for UNet model evaluation. Our analysis revealed that the segmentation accuracy was about 98.93%, and the mean intersection over Union (mIoU) was 90.53% with the category mean pixel accuracy (mPA) of 91.92% and the recall rate of 91.91%, respectively. These findings demonstrated that UNet model could be sufficient for CT image segmentation in this study.

**Fig. 4:**
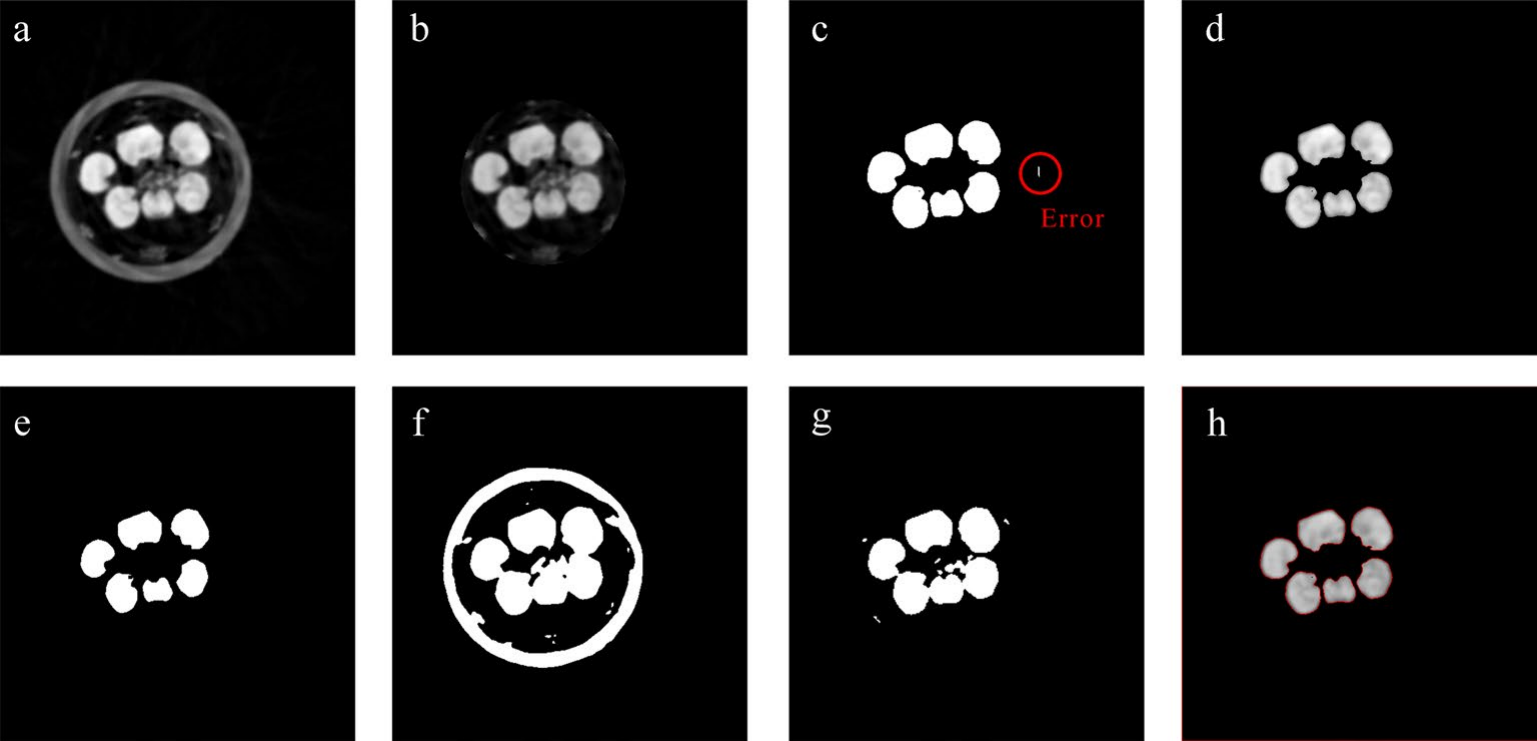
Example of slice image processing for detection of ROI. **a** Original CT slice image. **b** Holder-removed slice image. **c** UNet outputted image prediction of grains. Red circle highlights the error of UNet predicting. **d** Segmented image. **e** Assisted threshold segmentation image. **f** Direct threshold segmentation image without UNet. **g** Holder-removed direct threshold segmentation image without UNet. **h** Edge detection image

Finally, we encapsulated our pipeline in the Python language following the process steps shown in Fig. 1c–e. Firstly, a slice of CT images (Fig. 4a) was processed to remove the holder by CTAn software (Fig. 4b). Then, impurities such as dust or awns were eliminated during the UNet model segmentation process (Fig. 4c). The predicted results of UNet were set as a mask to segment the original images (Fig. 4d) and to obtain the ROI, followed by image binarization by the THRESH_OTSU + BINARY method provided by OpenCV for auxiliary segmentation (Fig. 4e). All processed images per spike were stacked and saved as NII type images for extraction of morphological traits. A slice of CT images contained the 2D coordinates of each pixel. After CT image processing, the resulting NII files contained 3D coordinates of each pixel, and a complete ‘virtual’ spike, stripped of other impurities (Fig. 1e).

### Extraction and validation of morphological features

**Fig. 5:**
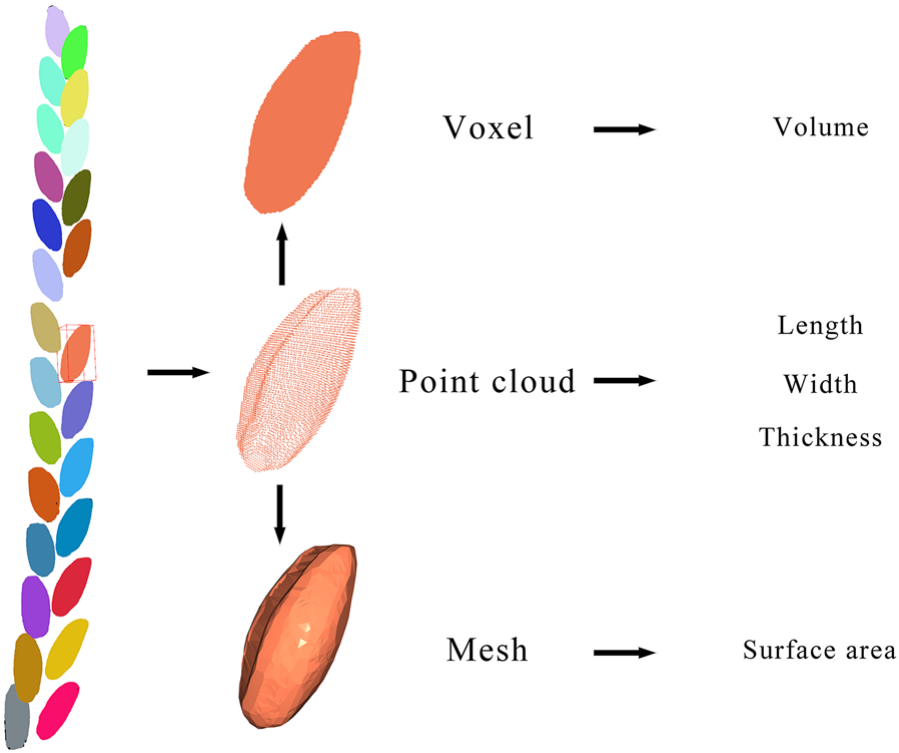
Example of spike point cloud image and process of conversion of grain images for phenotypic extraction. The colors in the figure are randomly adopted

The purpose of our pipeline was to extract biological traits from CT scans of barley spikes. To achieve the phenotypic trait extraction, we used a point cloud processing program Open3D to extract morphological traits from these stacked 3D images, and then generated a virtual spike and labeling individual virtual grains with random colors (Fig. 5). By applying these computer vision methods, we extracted the main spike-related traits from the perspective of intact barley spikes, including spike length, grain number per spike, total grain volume per spike and total grain surface area per spike. We also extracted grain length, width, thickness, volume, and surface area of each grain. The comprehensive definitions of the investigated phenotypes are shown in Additional file 3: Table S3.

**Fig. 6:**
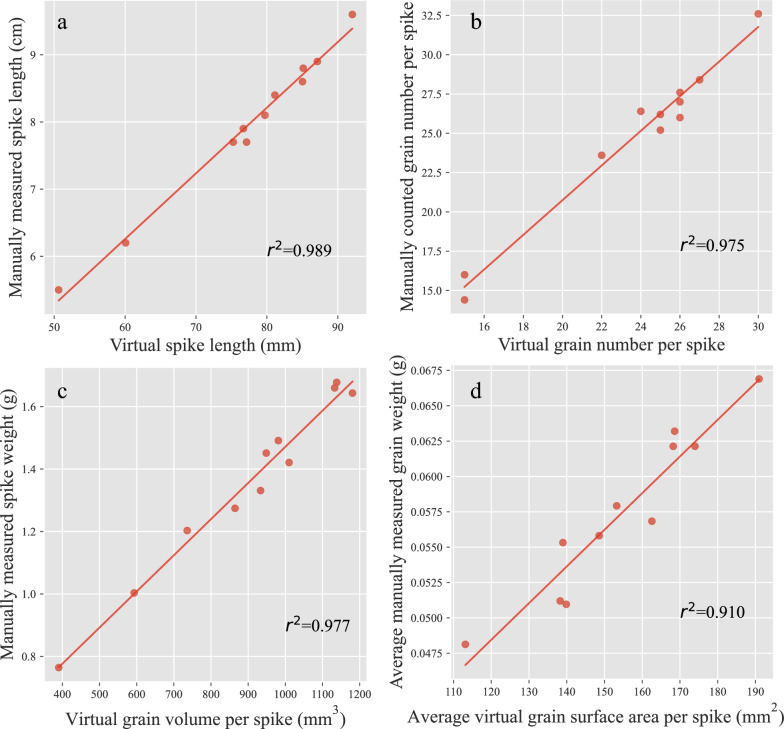
Validation of CT-based extraction of barley phenotypic traits. **a** Spike length. **b** Grain number per spike. **c** Relationship between spike weight and virtual spike volume. **d** Relationship between the spike weight and virtual spike surface area

To validate the accuracy of the virtual traits, we furthermore manually measured the spike-related and grain-related traits using rulers and seed analysis system. Results showed that the virtual spike length measured by the CT and DL imaging method was highly correlated with that of manual measurement (r^2^ = 0.989) (Fig. 6a). Similarly, the virtual grain number was also significant correlation with that of manual measurement, with the correlation coefficient of r^2^ = 0.975 (Fig. 6b). Simultaneously, the grain volume per spike was found to highly correlate with manually measured grain weight (r^2^ = 0.977) (Fig. 6c). However, the average virtual grain surface area per spike was found to be relatively slightly weaker correlation with the manually measured grain weight (r^2^ = 0.910) (Fig. 6d).

**Fig. 7:**
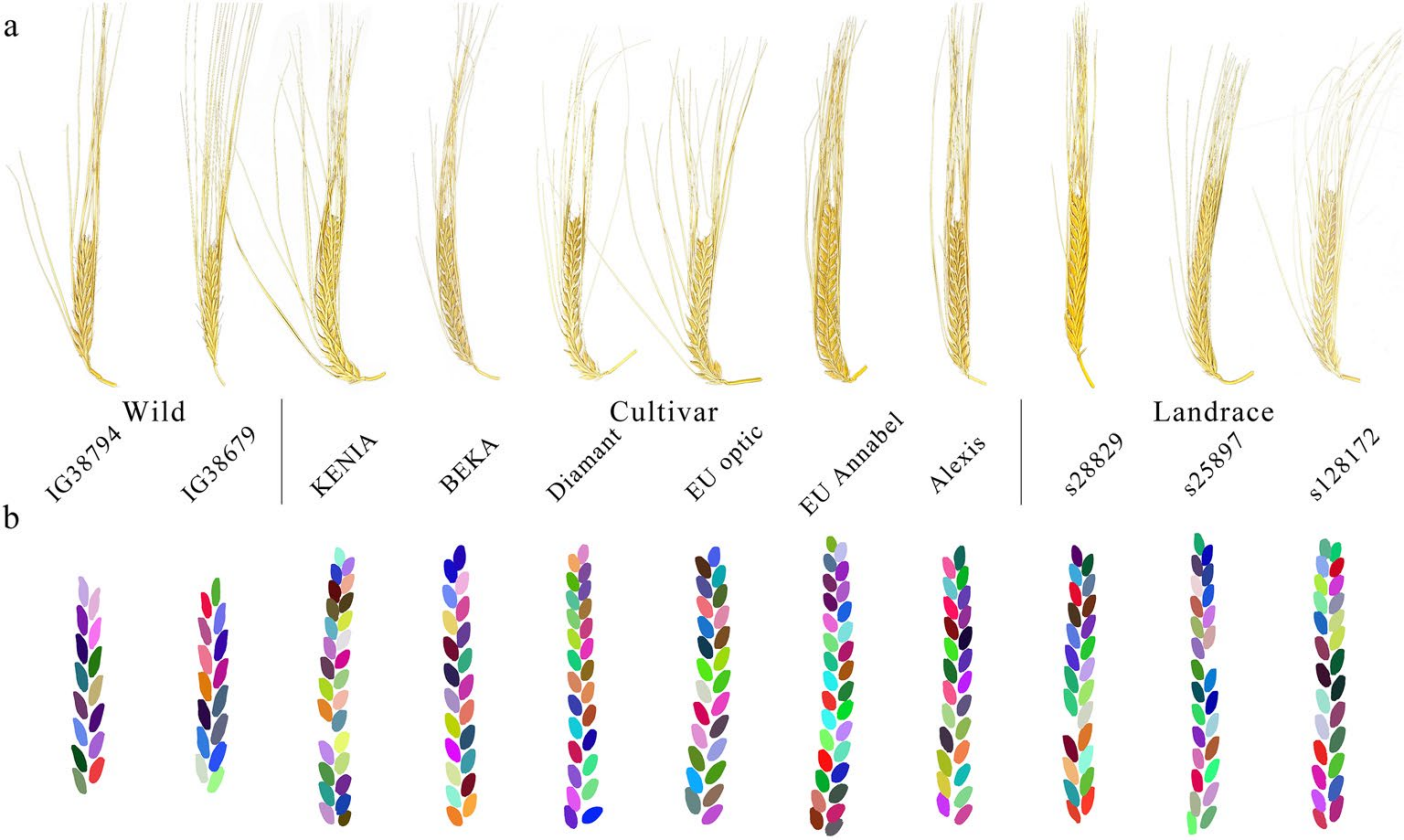
Spike images. **a** Optical images. **b** 3D reconstruction point cloud images with labeled virtual grains. The colors in the figures are randomly adopted

### Comparison of the spike morphometric traits among different barley

**Fig. 8:**
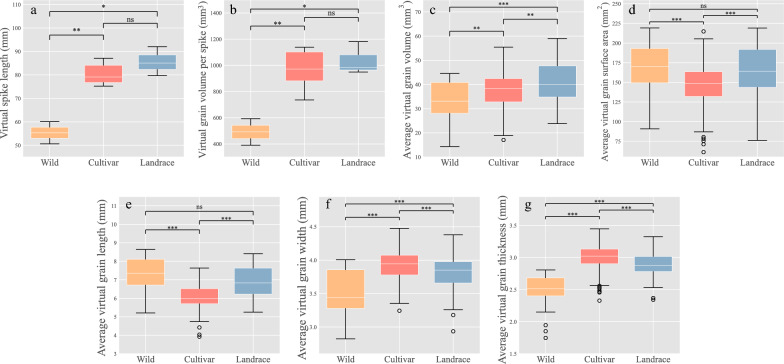
Comparison of morphological traits in wild barley, modern cultivar and landraces. **a** Virtual spike length. **b** Virtual grain volume per spike. **c** Average virtual grain volume. **d** Average virtual grain surface area. **e** Average virtual grain length. **f** Average virtual grain width. **g** Average virtual grain thickness. Ns, not significant; *, P < 0.05; **, P < 0.01; ***, P < 0.001

Based on the optical images and point cloud, we further compared the spike and grain-related traits among the three types of barley (Fig. 7). There was a distinct discrepancy in the virtual spike length among wild (55.3 ± 4.7 mm), cultivar (80.4 ± 4.5 mm), and landrace (85.6 ± 5.0 mm) (Fig. 8a). The virtual grain volume per spike also differed significantly, with wild barley having a smaller volume (491.8 ± 101.5 mm^3^) than cultivated barley (969.1 ± 143.5 mm^3^) and barley landrace (1,036.8 ± 102.6 mm^3^) due to the divergence in the number of grains per spike (Fig. 8b). The average virtual grain volume also demonstrated a substantial deviation among wild (32.8 ± 8.6 mm^3^), cultivar (37.5 ± 7.0 mm^3^) and barley landrace (40.9 ± 8.7 mm^3^) (Fig. 8c). The average virtual grain surface area of was 165.4 ± 33.7 mm^2^, 165.4 ± 32.6 mm^2^ and 145.6 ± 27.6 mm^2^ in wild balrey, barley landrace and cultivars, respectively (Fig. 8d). Apart from the average virtual grain length, other grain-related traits, including grain width and grain thickness also displayed significant differences among the three types (P < 0.001, Fig. 8e–g, Additional file 4: Table S4).

**Fig. 9:**
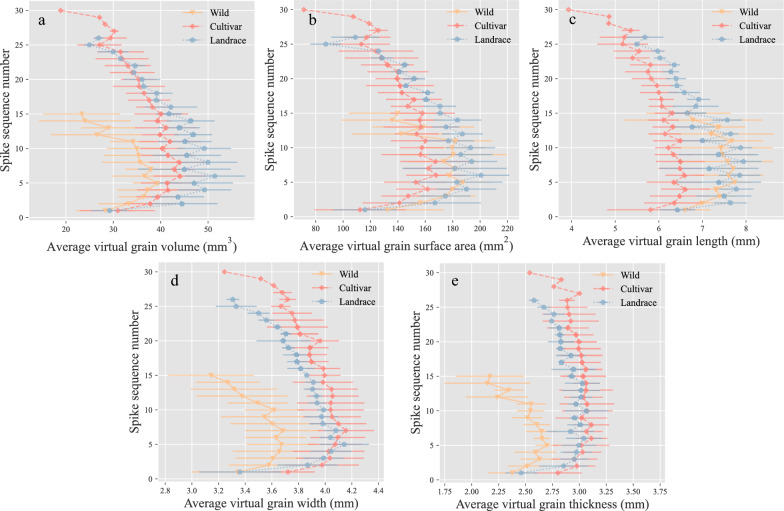
Distribution of grain morphological traits along the spike in three types of barley. **a** Average virtual grain volume. **b** Average virtual grain surface area. **c** Average Virtual grain length. **d** Average Virtual grain width. **e** Average Virtual grain thickness

The distribution of grain-related traits along the spike was further analyzed (Fig. 9). The grain number counted upwards from the bottom indicated the position of grain in the spike (Fig. 1f). As illustrated in Fig. 9, the distributions of average virtual volume, surface area, length, width, and thickness all peaked in the middle parts of the spike. The average virtual grain volume, width, and thickness distribution in the spike of wild barley was inferior to that of the other two types (Fig. 9a, d, e). The average virtual grain surface area along the spike showed a congruent trend for the three types (Fig. 9b). The average virtual grain length along the spike showed difference between cultivar and other two types (Fig. 9c).

## Discussion

### Feasibility of estimating spike morphometric traits from CT scan images

Utilizing the traditional phenotyping technique requires manual measurement in the field, leading to not only considerable labor and time investment, but also damage the specimens [11]. The proposed non-destructive method employs X-ray CT imaging and computer vision by image processing and trait extraction pipelines to characterize barley spikes and accurately quantify their morphometric features. The CT image segmentation pipeline robustly segmented the spike region of interest and analysis by application of the UNet model. The overall processing took only 4 min per spike to perform using a laptop (CPU-based speed: 3.30 GHz, RAM32GB). The extraction pipeline based on point cloud is highly efficient and fast, digitizing the morphological features in less than one minute using optimal point cloud cluster analysis parameters. The use of CT scanning technology is particularly noteworthy because it allows for non-destructive analysis of samples, preserving the seed’s integrity to sown after the experiment. The ability to capture high-quality images of plant structures in a non-invasive way can help researchers to better understand the structure and function of plant parts. Meanwhile, it can also be used to obtain some traits that can’t be easily accessed by normal manual measurement method, such as grain surface and volume.

### Combining X-ray CT scanning and DL modeling in plant phenotyping

Previous study has utilized a random forest machine learning framework to segment single X-ray CT plant leaf scans, which had limitations that some leaf tissues could not evenly segmented because the segmentation was not processed slice by slice [31]. We were able to surmount this dilemma by leveraging the DL UNet model to segment X-ray CT images better. In some basic workflows of crop spike X-ray CT scanning [14, 29], segmentation is performed only by binarization. As illustrated by Fig. 4f, g, the output of the direct binary threshold method produced a higher amount of noise, making it challenging to separate the virtual grains. Despite using a Deep Learning model for segmentation, errors may still arise (Fig. 4c); nevertheless, these can be circumvented through the implementation of a binary threshold. Subsequently, we applied the DL model for segmentation and then binarization to facilitate the production of more precise labeled CT images. As Fig. 4e demonstrated, we successfully extracted virtual grains from CT images without any image interference.

It is known that the image quality also hampered the precision. High-resolution images had not been incorporated into plant phenotyping pipelines due to the considerable time requirement for processing (greater than 1 h); however, it is conceivable that this data contained more useful details. If we refine the resolution (< 20 µm/pixel), we can get a more definite view when examining the internal structure of the spike, and our objective of utilizing the DL model may then switch to that of classifying grain components such as embryo, awn, and endosperm (Figs. 2c and 4a).

### Optimized 3D image processing for assessing spike-related traits

In order to quantify the spike- and grain-related features, the essential step is to extract grains from the virtual spike. A 2D-image-based method was proposed by Hughes, using CT slice image pixels for quantification of the morphology of grains and the calculation of the highest and lowest points in the z-axis [14]. 3D image processing procedures developed by Biao and Hu were comparable by ascertaining the neighboring voxels and isolating each grain [28, 29]. In this study, we exploited an improved 3D image processing strategy to pinpoint each grain with the help of point cloud dbscan clustering algorithm, relying solely on the codes provided by Open3D. Computing the attributes of grain can be done quickly at minimal expense on a personal computer in less than one minute.

By sorting the 11 barley accessions belonging to three types, we could more accurately discern the distinctions among them. The grains of wild varieties had a smaller average volume compared to cultivars and landraces, but displayed longer and slender in shape. It appears that the grains of modern barley varieties, despite their shorter and thicker structure, have higher volumes, larger numbers per spike, and greater yield. Interestingly, the virtual average surface area of each grain was analogous across all three types. Moreover, the distribution of grain traits along the spike revealed essential morphological specifics regarding barley spike architecture. The distribution of grain morphological traits along the spike indicated that grains in the middle region tend to be larger. Similar results were ascertained in previous studies on wheat spikes [29].

## Conclusions

This study offered a fast, efficient and non-destructive approach to quantitatively measuring the multitude of spike architecture in barley by integrating X-ray computed tomography (CT) and a deep learning model (UNet). It can efficiently and accurately investigate spike length, grains number per spike, volume and surface area of grains per spike, and grain length, width, and thickness in barley. Furthermore, it was able to determine the spatial distribution of grains along the spike. Based on the newly developed method, we systematically identified the discrepancies in spike morphology among wild barley, landrace and cultivars and the results showed that modern cultivated barley possesses shorter but more robust grains with bigger volumes and higher yield than wild barley and landrace. Our study highlights the potential of X-ray CT imaging and computer visioning in high-throughput phenotyping in barley, which will accelerate breeding of new varieties with enhanced yield.

## Methods

### Field experiment and plant materials

The barley materials were grown in a field trial of Northwest A&F University, in Yangling, Shaanxi province, China (108.08 E, 34.30 N). A total of 11 barley accessions were used in this study, including 2 wild barley, 3 landraces and 6 cultivars (Additional file 2: Table S2), which were planted using standard farm practices in the 2021–2022 cropping season. All treatments were replicated three times, and each was grown in a subdivided plot with 1.5 m in length and 0.6 m in width. All barley materials were sown on 24th October 2021 and harvested on 20th June 2022.

### X-ray CT scanning and image reconstruction

Spikes were scanned using a Skyscan 2214 instrument (Bruker Comp., Heidelberg, Germany). Each spike was placed in a plastic holder and a few spikes with weak stems were fixed by adhesive tape on a disposable plastic platform. The voltage and current of the scanner were set at 70 kV and 200 µA, respectively. The specimen platform rotation step was set at 0.9 degrees. Exposure time for each image was 550 ms, and 13,074 images were obtained at total (each number of varieties is listed in Additional file 2: Table S2). Each sample preparation and loading into the scanner took about 5 min, and scanning took about 10 min. 3D reconstruction was performed by NRecon (Version, 1.7.5) provided by the Bruker company (Heidelberg, Germany), outputting a set of transaxial slice 8-bit BMP images. The pixel size of each slice image was 75 to 95 µm (Additional file 1: Table S1). Reconstruction duration per slice took about 0.13 s.

### Computer hardware

After CT imaging, a DELL laptop computer with an Intel (i9 11980HK) chip with 8 cores, 16 threads of CPU, 32 GB of memory and an NVIDIA (RTX3080 Laptop) GPU was used for writing python scripts, preparation of datasets, training DL models, and morphological feature extraction.

### Image dataset preparation for UNet model training

Because spikes were scanned with a supporting holder, each reconstructed CT image was surrounded by a circle (Figs. 2c, 4a), which was removed by the Region of Interest (ROI) method using CTAn software (Bruker, Version, 1.18.8.0+). Three types of spikes (wheat cultivar D3, two-row barley variety s113095 and s17350) were chosen as identification prototypes, and 154 slice images were labeled for each one. Labelme (version, 5.0.1) was used to prepare labeled images.

### UNet architecture designation and model training

The original UNet model was used for segmentation of CT images in this study. All datasets and model architectures were prepared in Python (version, 3.9.7). Totally, 124 labeled images were used for training with 40 epochs and 4 batch sizes using the Pytorch package (version, 1.12.1 + cu116) with and 30 labeled images were used for testing. The parameter for the loss was established as BCEWITHLOGITSLOSS, culminating in the storage of the model with the least loss as the most advantageous model.

The performance of CT image segmentation accuracy analysis was evaluated using 4 metrics, such as precision, recall, mPA, and mIoU. These metrics are defined as follows:

1 $${\text{Precision}} = \frac{\text{TP}}{{\text{TP}} + {\text{FP}}}$$

2 $${\text{Recall}} = \frac{{\text{TP}}}{{\text{TP}} + {\text{FN}}}$$

3 $${\text{IoU}} = \frac{{{\text{TP}}}}{{{\text{TP}} + {\text{FP}} + {\text{FN}}}}$$

4 $${\text{mPA}} = \frac{{{\text{sum}}\left( {{\text{P}}_{{\text{k}}} } \right)}}{{\text{k}}}$$

5 $${\text{mIoU}} = \frac{1}{k + 1}\mathop \sum \limits_{k}^{i = 0} \frac{{{\text{TP}}}}{{{\text{TP}} + {\text{FP}} + {\text{FN}}}}$$

where TP, FP, and FN are the true positives, false positives, and false negatives, predicted by the segmentation model; k is the total number of categories; P_k_ is pixel accuracy per category.

### Slice image processing and stacking

A total of 10,353 transaxial slice images of 11 barley accessions were passed through the UNet model after resizing to 512 × 512 pixels, and outputting predicted images. The original images were also resized to 512 × 512 pixels using the OpenCV package (4.5.5.62). The predicted images were used as overlapping mask layers to segment the ROI of grains in the spike from the original images, followed by the image threshold segmentation method (THRESH_BINARY + THRESH_OTSU) provided by OpenCV to assist the segmentation process. After resizing to original pixel size, slice images were stacked to NII type via the SimpleITK package (version, 2.2.0) for easy preservation by classification and preparation for the next step.

### Extraction of plant phenotypic traits from image stacks

The 11 image stacks were visualized by 3D Slicer software (version 5.0.3) (and named ‘virtual’ spikes. The NII type files were loaded into the Python environment by the Nibabel package (version, 2.5.1) and the watershed method was used for contour detection by OpenCV prior to transforming all array data to point cloud data by the Open3D package (0.16.0). The point cloud data of virtual spikes was processed by the dbscan clustering algorithm (parameter, eps = 3–7, Additional file 1: Table S1) by Open3D to segment each grain for counting, and surface reconstruction using the Alpha shapes method (alpha = 12) to calculate the surface area of grains from the watershed method results. The length, width and thickness of grains and spikes were calculated by the point cloud oriented bounding box. The grain volume and surface area were calculated using formulae as follows:

6 $$V = \frac{{nP^{3} }}{{10^{9} }}\;mm^{{3}}$$

7 $$S = \frac{{mP^{2} }}{{10^{6} }}\;mm^{{2}}$$

where* V* is calculated volume; *n* is the number of voxels; *P* is pixel size (um); *S* is calculated surface area; *m* is the area of mesh surface; *mm* is millimeter.

### Data analysis and validation of the CT-based morphological method

Spikes of 11 barley accessions were selected following feasible results of segmentation of virtual spikes. The number of grains and the length of spikes were manually ascertained and compared with the data obtained from the CT-based method. The weight of spikes was gauged by balance and their correlation with the calculated volume and surface area of virtual spikes were determined.

### Statistical analysis

The Matplotlib package (version, 3.5.2) was used for plotting, and the Scipy package (version, 1.9.0) was used for analysis as well as statistical analysis was conducted with Python. Correlation analysis was performed using the ‘corrcoef’ function in the Numpy package (version, 1.22.3). *t* test was used to compare the differences among three barley types in the Scipy package.

### Supplementary Information

**Additional file 1: Table S1.** Image resolution and point cloud clustering parameter of CT images.

**Additional file 2: Table S2.** Detailed descriptions of the experimental barley materials.

**Additional file 3: Table S3.** Detailed descriptions of phenotype definition.

**Additional file 4: Table S4.** Comparison of the spike morphometric traits among three type barley materials.

## Data Availability

All code and datasets pertaining to deep learning segmentation training, predicting and barley spike traits extraction is open-sourced on Github at https://github.com/zerosky010/CT_barley_spike_detection.

## Abbreviations

CT: Computed tomography
ROI: Regions of interest
DL: Deep learning
ML: Machine learning
RGB: Red–green–blue imaging
ChlF: Chlorophyll fluorescence
FCN: Fully Convolutional Network
2D: Two-dimensional
3D: Three-dimensional
MiB: Mebibyte
GiB: Gibibyte
IoU: Intersection over union
mPA: Mean pixel accuracy
